# Obstacles and Satisfaction to Balance Between Family Life and Medical Career Among Saudi Women Doctors

**DOI:** 10.7759/cureus.38759

**Published:** 2023-05-09

**Authors:** Bayan S Alahmadi, Lama S Alahmadi, Faris M Eltoum

**Affiliations:** 1 Family Medicine, Ministry of Health, Madina, SAU; 2 Medicine and Surgery, Al-Rayan Colleges, Al Madinah, SAU; 3 Medicine, Al-Rayan Colleges, Al Madinah, SAU

**Keywords:** cross-sectional, challenging of balance, family life, female, satisfaction, saudi arabia, madinah, career, physicians

## Abstract

Background: Besides their medical career obligations, female physicians traditionally have assumed responsibility for raising families and maintaining the household. Finding an acceptable balance between their career and family life is challenging.

Objective: The study aimed to discover the obstacles and the relationship between the barriers/factors with the satisfaction in balancing career and family life.

Methodology: A cross-sectional study that analyzed data from Saudi female physicians. The study included 165 female physicians from the six Ministry of Health hospitals in Al-Madinah Al-Munawarah; 65 were specialists and consultants, and 100 were general practitioners and residents. The data were collected from October until the end of November 2022 through a semi-structured, self-administered questionnaire gathered by convenience sampling. The data were collected and analyzed with SAS software.

Results: The study's main findings include a satisfaction rate in balancing career and family life among the studied female physicians, which was low at 15.7%. In comparison, female physicians unsatisfied with such balancing were 38.2%. The effect of family responsibilities on career choice was nearly equal, where 50.3% of the studied female physicians affected them. There was a statistically significant difference regarding the satisfaction in balancing career with family life by their specialty; female surgeons and gyn/obs physicians found a higher percentage of unsatisfaction, whereas, among family medicine physicians, the least rate of unsatisfaction was found (P-value < 0.01). At 80% of the studied physicians suggested providing childcare centers as the main solution to their difficulties and obstacles; also, 46.5% suggested taking more days off of maternity leave. Transportation difficulties, however, represented the lowest type of difficulty, 12.7%.

Conclusion: The present study has revealed several obstacles facing female physicians that negatively impact relationships with their families.

## Introduction

Female employment in Saudi Arabia nowadays shows a great expansion as a modern phenomenon, especially in the healthcare sector. However, it was much easier in the past when the majority of healthcare workers were men, and their wives were at home taking care of their children and running the household [1]. Working in the health sector is usually characterized by long working hours, on-calls and post-calls among doctors, and frequently rotating shift work in some specialties, such as the emergency department. This makes the priority for the patient’s welfare above the family, social lives, and personal needs [1,2]. As women entered the medical field in increasing numbers, the tensions between career and family life became more prominent. In 1981, Saudi women doctors constituted 4.4% of professionally active Saudi doctors. In 1997, this number reached 20.36% [3]. The last estimated numbers done in King Abdul-Aziz Medical City, Riyadh 2013, showed markedly increased numbers (34.89%). Jeddah has the majority of female doctors compared to other cities [4]. In Saudi Arabia, several studies have propped this subject in different regions. In the Al-Hassa region, a cross-sectional study about maternal employment and maternity care showed that working mothers-initiated care late in pregnancy and subsequently attended fewer visits. They had more cesarean sections, preterm deliveries, and low birth weight infants in the index pregnancy [5]. These adverse effects were more prominent with unfavorable working conditions. Recently, another cross-sectional study was conducted on 174 Saudi female physicians in King Abdul-Aziz Medical City in Riyadh and found that more than half of them 56.3% reported discrimination from colleagues because of their marital status. The study also revealed that 43.1% of them were unsatisfied in balancing their career and family life, about half of them aunts 51.7% thought their work has a negative impact on their relationship with their spouses/children, and 51% of them reported that care of children and possibilities of combining work and responsibilities for children and family had been of great importance in their choice of specialty. Most of the above-mentioned studies, however, were interested in finding out the levels of burnout and satisfaction among the studied female physicians, and a few studies were concerned with finding out the common difficulties and obstacles and their relation to balancing family life among female physicians. The present study aimed to find out the obstacles in balancing between career and family life, and to assess the relationship between the barriers/factors (demographic factors, family responsibilities, career obligations) with the satisfaction in balancing between career and family life [6].

## Materials and methods

Study design

The current study is an observational cross-sectional study of Saudi female physicians working in the hospitals of the Ministry of Health in Madinah, with a total number of 279 physicians. All female Saudi physicians will be eligible to participate in this study and non-Saudi female physicians will be excluded. Also, female pharmacists, technicians, nurses, and other staff clerks will not be invited. The study was carried out from the start of October until the end of November 2022.

Tool of the study and data collection

The data were collected using a self-administered questionnaire submitted by the female physicians in their departments. The questionnaire was divided into four sections. The first section included personal data (age, marital status, specialty, academic level, and type of job). The second section included information about the impact of family responsibilities on careers, such as the number of children, the spouse's employment status, the duration of work gaps due to family or social responsibilities, the effect of specialty choice on family responsibilities, discrimination or negativity from colleagues because of marital status, and discrimination or negativity from society because of work as a physician. The third part included data about career obligations, and the fourth part included data about the obstacles faced by the studied physicians and their suggested solutions.

Statistical analysis

The statistical analysis system (SAS version 9.1; SAS Institute, Cary, NC) was used to analyze the data collected from the 165 female physicians. Physicians' characteristics were tabulated, where categorical variables were presented by their frequency number and percent, and continuous variables were presented by their mean ± SD. Physicians' satisfaction in balancing career with family life and other impacts of career obligation were compared by their personal characteristics using Chi-square and Fischer exact as appropriate. P-value ≤ 0.05 was considered an indicator of a statistically significant difference.

## Results

A total of 165 Saudi female physicians were analyzed to find out the different obstacles that Saudi female physicians face in balancing their medical career with their own family life in Al-Madinah City and to assess the relationship between the barriers/factors (demographic factors, family responsibilities, career obligations) and the satisfaction in balancing career and family life.

**Table 1:** Personal characteristics of the Saudi female physician

Characteristics*	N= 165
Age in years; mean ± SD	31.3 ± 4.5
Age in years	N= 165
25 - < 30	90 (54.5%)
30 - < 35	47 (28.5%)
35 - < 40	13 (7.9%)
≥ 40	15 (9.1%)
Marital status	N= 165
Single	7 (4.2%)
Married	106 (64.2%)
Divorced and widow	52 (31.6%)
Number of children**	N= 165
No children	37 (28.9%)
2 or 1	64 (50%)
≥ 3	27 (21.1%)
Academic level	N= 165
Consultant	16 (9.7%)
Assistant consultant	49 (29.5%)
Resident	92 (55.8%)
General physician	8 (4.9%)
Specialty	N= 165
Family medicine	50 (30.3%)
Internal medicine	17 (10.3%)
Pediatrics	32 (19.4%)
Gyn/obst	25 (15.2%)
Surgery	5 (3%)
Others	36 (21.9%)
Type of jobs	N= 165
Clinics only	67 (40.6%)
On calls	91 (55.2%)
Shifts	7 (4.2%)
Husband's employment	N= 165
Physicians	32 (27.4%)
Non physicians	133 (80.6%)

Table 2 shows the distribution of the Saudi female physicians by impact of family responsibilities. About two-thirds of the studied female physicians reported that there was an interruption in their career because of their family responsibilities while one-third reported such interruption, of them 16.4% interrupted for less than one year, and 2.4% interrupted for more than five years. The effect of family responsibilities on career choice was nearly equal where half of the studied female physicians answered “yes” and the other half answered “no.” Discrimination at work because of marital status was reported by 38.2%, and discrimination in society because of work was denied by 57.5% as shown in Table 2.

**Table 2:** Distribution of the Saudi female physicians by impact of family responsibilities

Item	N=165 (%)
Duration of interruption because of family responsibilities	
< 1 year	110 (66.7%)
1-2 years	27 (16.4%)
3-5 years	18 (10.9%)
> 5 years	6 (3.6%)
Family responsibilities affecting choice of career.	
Yes	83 (50.3%)
No	82 (49.7%)
Discrimination at work because of marital status	
Yes	63 (38.2%)
No	102 (61.8%)
Discrimination at society because of work	
All the time	25 (15.2%)
Sometimes	45 (27.3%)
Never	95 (57.5%)

Table 3 shows the distribution of the studied female physicians by the impact of career obligations on family life. The number of hours worked per week varied among the studied female physicians, with 60% reporting to work from 40-60 hours weekly and only 16.4% reporting to work more than 60 hours per week. Satisfaction in balancing a career with family life was found in 15.7% of the studied female physicians and dissatisfaction was reported by 38.2% of them and the remaining females were neutral on this point. More than one-third of the studied females 36.3% reported a negative impact of their careers on their spouse's relationship, half of them reported a negative impact on their children's relationship, and 30.3% of them reported that their careers had a negative impact on their children's performance. The daily house duties were done completely 100% by only 12.15% of the studied female physicians, while 27.3% of them reported performing less than 25% of their house duties. Transportation difficulties were reported by 31.5% of the studied female physicians.

**Table 3:** Distribution of the Saudi female physicians by impact of career obligation on family life

Item	N=165 (%)
Working hours/week	
< 40 hours	37 (22.4%)
40-60 hours	101 (61.2%)
> 60 hours	27 (16.4%)
Satisfaction in balancing career with family life	N (%)
Satisfied	26 (15.7%)
Neutral	76 (46.1%)
Unsatisfied	63 (38.2%)
Negative impact on spouse relationship	N (%)
Yes	60 (36.3%)
No	31 (18.9%)
Neutral	74 (44.8%)
Negative impact on children relationship	N (%)
Yes	81 (49.1%)
No	11 (6.7%)
Neutral	73 (44.2%)
Negative impact on children performance at school	N (%)
Yes	50 (30.3%)
No	27 (16.4%)
Neutral	88 (53.3%)
Daily household duties in percentage	N (%)
100%	20 (12.1%)
75%	25 (15.2%)
50%	36 (21.8%)
25%	39 (23.6%)
< 25%	45 (27.3%)
Difficulties in hospital transportation	N (%)
Yes	52 (31.5%)
No	56 (33.9%)
Sometimes	57 (34.6%)

Table 4 displays the distribution of the studied female physicians' satisfaction in balancing career with family life by their marital status. There was no significant difference regarding the satisfaction in balancing career with family life by the studied female physicians' marital status (p=0.48). However, the percentage of satisfaction was higher among not married females 20.3% compared to 13.2% among married females. Also, the unsatisfied percentage was higher among married females at 39.6% compared to that among not married females at 35.6% as shown in Table 4.

**Table 4:** Distribution of the Saudi female physicians and their satisfaction in balancing career with family life by their marital status

Satisfaction	Married (n= 106)	Not married (n= 59)	P-value
Satisfied	14 (13.2%)	12 (20.3%)	0.48
Neutral	50 (47.2%)	26 (44.1%)	
Unsatisfied	42 (39.6%)	21 (35.6%)	

Table 5 presents the distribution of the studied female physicians' satisfaction in balancing their career with family life by their job type. The satisfaction was higher among physicians with clinics only work 19.3% compared to 13.2% and 14.3% among those physicians with on-calls and shift work, respectively. However, the percentage of unsatisfied females was higher among on-calls work females 47.3%. Although of these observed variations in satisfaction by job type, there was no statistically significant difference (p=0.12).

**Table 5:** Distribution of the Saudi female physicians and their satisfaction in balancing career with family life by their job type

Satisfaction	Clinics only	On-calls	Shifts	P-value
N	(n= 67)	(n= 91)	(n= 7)	0.12
Satisfied	13 (19.3%)	12 (13.2%)	1 (14.3%)	
Neutral	36 (53.7%)	36 (39.5%)	4 (57.1%)	
Unsatisfied	18 (26.8%)	43 (47.3%)	2 (28.6%)	

Table 6 shows the distribution of the studied female physicians' satisfaction in balancing career with family life by their academic level. Consultants and specialists showed a higher percentage of satisfaction 18.5% compared to that reported among residents and general practitioners 14%, although not significant (p = 0.69). Unsatisfied percentage was higher among residents and general practitioners 40% compared to those consultants and specialists 38.3%.

**Table 6:** Distribution of the Saudi female physicians and their satisfaction in balancing career with family life by their academic level

Satisfaction	Consultants and Specialists	Residents and general physicians	P-value
N	(n= 65)	(n= 100)	0.69
Satisfied	12 (18.5%)	14 (14%)	
Neutral	30 (46.2%)	46 (46%)	
Unsatisfied	23 (38.3%)	40 (40%)	

Table 7 displays the distribution of the studied female physicians their satisfaction in balancing career with family life by their specialty. There was a statistically significant difference regarding the satisfaction in balancing career with family life by the studied female physicians' specialty (p=0.01). The higher percentage of unsatisfaction was found among female surgeons 60%, followed by gyn/obs physicians 56%, and pediatrics and internal medicine physicians 53.1%. The least percentage of unsatisfaction among the studied females was found among family medicine physicians 22%.

**Table 7:** Distribution of the Saudi female physicians and their satisfaction in balancing career with family life by their specialty

Satisfaction	Family medicine	Internal med. and pediatrics	Gyn/obs	Surgeons	Others	P-value
N=165	(n= 50)	(n=49)	(n= 25)	(n= 5)	(n= 36)	0.01*
Satisfied	9 (18.0%)	4 (8.2%)	3 (12%)	1 (20%)	9 (25%)	
Neutral	30 (60%)	19 (38.7%)	8 (32%)	1 (20%)	18 (50%)	
Unsatisfied	11 (22%)	26 (53.1%)	14 (56%)	3 (60%)	9 (25%)	

Table 8 presents the negative impact on spouse and children relationship and performance among the studied female physicians by their academic level. The career negative impact on spouse relationship was higher among consultants and specialists 46.2% compared with that among residents and general practitioners 30%, although not significant. Also, not significant higher percentage of negative impact on children relationship was observed among consultants and specialists 56.9%. A statistically significant difference was detected regarding the negative impact of career on the children's performance at school with the higher percentage was found in consultants and specialists 49.6% compared to only 21% among residents and general practitioners (p=0.01).

**Table 8:** Negative impact on spouse and children relationship and performance among the Saudi female physicians by their academic level

Satisfaction	Consultants and Specialists	Residents and general physicians	P-value
Negative impact on spouse relationship	(n= 65)	(n= 100)	0.08
Yes	30 (46.2%)	30 (30%)	
No	12 (18.5%)	19 (19%)	
Neutral	23 (35.4%)	51 (51%)	
Negative impact on children relationship	(n= 65)	(n= 100)	0.18
Yes	37 (56.9%)	44 (44%)	
No	5 (97.7%)	6 (6%)	
Neutral	23 (35.4%)	50 (50%)	
Negative impact on children performance at school	(n= 65)	(n= 100)	0.01*
Yes	29 (49.6%)	21 (21%)	
No	12 (21.5%)	13 (13%)	
Neutral	22 (33.9%)	66 (66%)	

Table 9 presents the negative impact on spouse and children relationship and performance among the studied female physicians by their job type. The career negative impact on spouse relationship was higher among “on-calls” job physicians 37.4%, and the lowest among “shifts” work physicians 28.5%, although not significant. The negative impact of career on children relationship, however, was higher among “clinics only” physicians 55.2%, followed by “on-calls” physicians 45.1% and the lowest among “shifts” work physicians 42.9%. The negative impact of career on children's performance at school showed the higher percentage among “on-calls” and “clinics only” physicians with the reported percentages were 31.9% and 30%, respectively.

**Table 9:** Negative impact on spouse and children relationship and performance among the Saudi female physicians by their job type

Satisfaction	Clinics only	On calls	Shifts	P-value
Negative impact on spouse relationship	(n= 67)	(n= 91)	(n= 7)	0.46
Yes	4 (35.8%)	34 (37.4%)	2 (28.5%)	
No	14 (20.9)	14 (15.4%)	3 (43%)	
Neutral	29 (43.3%)	43 (47.2%)	2 (28.5%)	
Negative impact on children relationship	(n= 67)	(n= 91)	(n= 7)	0.41
Yes	37 (55.2%)	41 (45.1%)	3 (42.9%)	
No	6 (9%)	5 (5.5%)	0 (0%)	
Neutral	24 (35.8%)	45 (49.4%)	4 (57.1%)	
Negative impact on children performance at school	(n= 67)	(n= 91)	(n= 7)	0.22
Yes	20 (30%)	29 (31.9%)	1 (14.3)	
No	15 (22.4%)	12 (13.1%)	0 (0%)	
Neutral	32 (47.6%)	50 (55%)	6 (85.7%)	

Table 10 presents the distribution of the studied female physicians by difficulties they face in balancing career and family life. The difficulties facing the studied female physicians were pregnancy 75.2%, children 73.9%, and household duties 73.3%. Educational level and work situation of husband was reported by 76 of the studied female physicians 46.1%. Transportation difficulties, however, were representing the lowest type of difficulties faced by the studied female physicians where only 12.7% of them reported this difficulty.

**Table 10:** Distribution of the Saudi female physicians by difficulties they face in balancing career and family life

Item	N=165 (%)
		Children	122 (73.9%)
Household duties	121 (73.3%)		
		Pregnancy	124 (75.2%)
Educational level and work situation of husbands	76 (46.1%)		
		Transportation to hospital	21 (12.7%)

Table 11 shows the solution suggested by the studied female physicians to make a balance between career and family life. The majority of the studied physicians 80% suggested providing childcare and medical center as the main solution to their difficulties and obstacles facing them. More than two-thirds 71.1% suggested flexibility of work, 58.8% of them suggested more days off of married women, 46.5% suggested more days off of maternity leave, 46.1% suggested decreasing work hours, and 33.9% of the studied female physicians suggested strong monitoring program as a solution of obstacles they face.

**Table 11:** Distribution of the Saudi female physicians by their suggested solution to make balance between career and family life

Item	N=165 (%)
		Providing childcare and medical centers	132 (80%)
Flexibility of work	118 (71.1%)		
		Decreasing working hours	76 (46.1%)
Strong monitoring program	56 (33.9%)		
		More days off of married women	97 (58.8%)
More days off of maternity leave	76 (46.5%)		

## Discussion

The present cross-sectional study has analyzed data from Saudi female physicians to find out the different obstacles among the studied physicians in balancing their medical careers with family life. The study included 165 female physicians from the six Ministry of Health hospitals in Al-Madinah City; 65 of them were specialists and consultants, and 100 were general practitioners and residents.

The impact of family responsibilities on medical career has shown that 33.3% of the studied physicians report medical career interruption because of their family responsibilities, of them 16.4% were interrupted for less than one year and 2.4% were interrupted for more than five years. Striking a balance between personal and professional obligations is difficult for all physicians. For women, however, it is made more challenging by their domestic obligations [6].

The study findings revealed that the effect of family responsibilities on career choice was nearly equal where half of the studied female physicians answered “yes” and the other half answered by “no.” In previous similar studies, particularly in Saudi Arabia, family responsibilities appeared to have no role in career choice, and other factors appeared to have the upper hand in the choice. The recent Saudi study on 126 male and 247 female physicians showed lifestyle was the most important factor influencing the choice of a future specialty among all participants. Lifestyle was cited by 168 participants (44.7%) as the factor that had the most influence on their decision regarding a future specialty, followed by subspecialty preferences by 49 individuals (13%) and clinical rotation experience by 43 participants (11.4%). Also, according to the logistic regression in that study, subjects' sex appeared to be the most important predictor of career choice among the studied male and female physicians [7]. Also, the linear regression analysis, in a study conducted in Al-Madinah City, Saudi Arabia, on 170 interns and residents working at Al-Madinah hospitals, showed that specialty character to be the most important predictor among the studied female physicians. In another Saudi study, however, 51% of the 174 female doctors studied in King Abdul-Aziz Medical City in Riyadh said that taking care of children and being able to work and take care of their families at the same time was very important in their choice of specialty [8].

In the present study, discrimination at work because of marital status was reported by 38.2%, and discrimination in society because of work was denied by 57.5%. A much higher rate of discrimination was reported in a cross-sectional study conducted on 174 Saudi female physicians in King Abdul-Aziz Medical City in Riyadh. The study results found that more than half of female physicians (56.3%) reported discrimination from colleagues because of their marital status. Physician surveys have consistently found that the majority of discrimination was related to racial and ethnic status. Some studies have reported that racial/ethnic minority physicians state they experience racial/ethnic discrimination in the workplace (69-71). Other studies have suggested that workplace discrimination may contribute to disparate career outcomes among racial/ethnic minority physicians such as lower rates of promotion and career satisfaction when compared with nonminority physician peers with similar productivity (72-75). In our Muslim communities, however, such type of discrimination was not reported at all.

The career impact factors in the present study have revealed that 60% of the studied female physician work from 40 to 60 hours weekly and only 16.4% reported to work more than 60 hours per week. Long hours and responsibilities are known to put the needs of patients ahead of personal needs and family responsibilities. This is seen as a challenge, especially for female doctors who are in practice [9].

Satisfaction with balancing career with family life was found in 15.7% of the studied female physicians, while female physicians unsatisfied with such balancing were 38.2%. Stratified analysis by personal characteristics has only revealed statistically significant differences regarding the satisfaction in balancing career with family life by physicians' specialty (p=0.01). The higher percentage of unsatisfied females was found among female surgeons (60%), followed by gyn/obs physicians (56%), and pediatrics and internal medicine physicians (53.1%). The least percentage of unsatisfied females was found among family medicine physicians (22%). A similar rate was also reported in the previous Saudi study conducted in King Abdul-Aziz Medical City in Riyadh, where 43.1% of the studied 172 female physicians were unsatisfied with balancing their career and family life. A much higher rate of unsatisfaction was reported in a previous study conducted in the teaching Hospitals of Karachi, Pakistan, where 68% of the working doctors were not satisfied and there was very little difference among the studied male and female doctors regarding their job satisfaction scores [10].

In contrast to the above-mentioned findings, a large survey included 1,380 female physicians, however, reported that most women were satisfied with their career as an emergency physician, 492 (35.5%) very satisfied, 610 (44.0%) satisfied, 154 (11.1%) neutral, 99 (7.1%) not satisfied, and 31 (2.3%) very unsatisfied. Less job satisfaction makes people both inside and outside of work feel stressed [11]. This stress can cause burnout, illness, and trouble in relationships (78, 79). All of these things are important because women doctors are more likely than men to report drug problems or commit suicide [12].

The daily house duties were done completely (100%) by only 12.1% of the studied female physicians, while 27.3% of them reported to perform less than 25% of their house duties. In a previous Canadian study, Heins et al. surveyed 87 female physicians. At least 76% of the respondents did all the cooking, shopping, childcare, and money management. Nearly all had responsibility for most household tasks, and 30% had no domestic help. Eighty-four percent reported that they were satisfied with the household arrangements. However, when asked more specifically whether they felt there were too many demands on their time and energy in looking after a job and a house, most said yes. Nearly 50% quoted role conflict as the reason, 25% felt that child care was the one important reason, while 65% mentioned homemaking and the mothering role. When asked about how change could be achieved, more than one-third mentioned better household help, 42% blamed themselves for excessive demands on time and energy, and only 5% thought husbands and children could help more [13]. Furthermore, the Ontario survey of 135 female physicians revealed that the responsibility for ensuring that the children were looked after fell mostly to the woman [13]. One-third stated they could not make definite future professional plans because of the demands made by their husbands and/or children. One-third had no domestic help. In that study, the most presenting problems among the studied physicians were long hours and conflict between family and house duties and career [13].

The study findings on the impact of career obligation on the studied female physicians' relationship with their spouse and children have revealed that more than one-third of the studied females (36.3%) have reported a negative impact of career on spouse relationships and a half of them reported a negative impact on children relationship, and 30.3% of them reported that their career to have a negative impact on their children performance. Stratified analysis by the studied physicians' title, job type, and specialty did not show any statistically significant difference, although percentage of negative impact was higher among surgeons, gyn/obs, and pediatrics and internal medicine physicians. Professional obligations among physicians often conflict with personal and family life, and it has an effect on the relationship with physicians' spouses and children (81, 82). A similar result was also reported in the previous Saudi study, which reported that 51.7% of the studied 174 female physicians think their work has a negative impact on their relationship with their spouses/children, and 51% of them reported that care of children and possibilities of combining work and responsibilities for children and family had been of great importance in their choice of specialty [14].

In the United States, one of the first studies to examine this subject was conducted. In a study that was published over 45 years ago, doctors were shown to have significantly higher divorce rates and lower marital quality ratings than workers in other professions [15]. Nevertheless, the study's generalizability was constrained by its small and unrepresentative sample size. A later, larger survey of 1,118 Johns Hopkins University medical graduates reported cumulative divorce rates of 29%, with higher rates among psychiatrists (50%) and surgeons (33%). However, this study was also limited by its analysis of physicians from a single institution [15]. There were several studies have evaluated marital quality among physicians and examined factors associated with self-reported marital quality (85, 86). Among these factors are working hours, physician specialty area, practice setting, spousal occupation, and presence of children.

The present study has assessed the most common obstacles female physicians face in balancing career and family life, and their suggestions to solve them. The difficulties facing the studied female physicians in this study were pregnancy (75.2%), children (73.9%), household duties (73.3%), and the educational level and work situation of the husband (46.1%).

Participating doctors have dealt with challenges in various ways, such as opting to have a small family size (82.3%), hiring a nanny or housekeeper (76.3%), and splitting the family income (62.0%). The varied and changing demands placed on physicians should be considered and accommodated in public expectations, workforce policy, and physician training programs [14].

The suggestive solutions mentioned by the studied female physicians include childcare and medical center (80%), and more than two-thirds (71.1%) suggested flexibility of work and more days off. In the previous Saudi study (3), the suggested solutions reported by the participating female physicians were limited in providing childcare in medical centers (79.9%), and more days off for maternity leave (75.3%) were the most reported solutions suggested. However, in the Egyptian study, the participating doctors dealt with difficulties in various ways, including choosing to have a small family size (82.3%), hiring a nanny or maid (76.3%), and splitting the family income (62.0%). Public expectations, workforce policy, and physician training programs should all take into account and adapt to the many evolving demands of physicians (63). The flexibility afforded in practice is one of the reasons more women than males enter the field of medicine, as established in earlier studies, to assist women in juggling their many obligations [16].

Among solutions mentioned by most of the female physicians in the present study to improve the balance between career and family life is providing more days off for maternity leave. The American Academy of Physicians says that one parent should be the infant's primary caretaker for at least four months and that maternity leave should start at least two weeks before the expecting mother's due date [17]. The length of paid maternity leave in Canada is 20 weeks for doctors (including residents) [18]. Employers typically give mothers a minimum of 12 weeks of paid leave in the industrialized world [19]. However, according to government rules and regulations, paid maternity leave days in Kingdom of Saudi Arabia must finish no later than 40 days following the day of delivery. According to these findings, more legislative and policy changes, concerning the duration of maternity leave, have to be taken by the policymakers to protect practicing female physicians in the Kingdom.

The strength of this study includes that the present study has used data from a relatively large sample size of Saudi female physicians from all ministries of health hospitals in Al-Madinah (i.e., it is not a single institution study). Also, it is included female physicians of different specialties. The used study questionnaire was validated by three family medicine consultants. This study is considered to be the first in Al-Madinah to study to probe the obstacles and solutions to balancing family life with a medical career among Saudi female physicians.

This study has also a number of limitations. Self-selection bias may have been a limiting factor in this study because those subjects who chose to participate may be more exposed to obstacles than others. However, because of the small refuse rate encountered in this study, the large number included out of the total number of female physicians in the studied hospital, this factor appeared to have no role in the study findings.

## Conclusions

The present study has revealed several obstacles facing female physicians in the studied six Ministry of Health hospitals in Al-Madinah City. There have been negative impacts of these obstacles on the relationship between the studied female physicians and their spouses and children. The most important solutions mentioned by female physicians in this study were providing childcare centers at the hospitals, the flexibility of work, and more days off for maternity leave. According to available data about the paid maternity leave days, that did not exceed 40 days after the date of delivery, as per rules and regulations of the Saudi government, legislative and policy changes have to be taken by the policymakers to protect practicing female physicians in the Kingdom.
